# Regional cerebral perfusion during arch repair in infants: there is always room for improvement

**DOI:** 10.1093/icvts/ivad008

**Published:** 2023-01-28

**Authors:** Mirela Bojan, Emre Belli

**Affiliations:** Department of Pediatric and Congenital Heart Disease, Marie Lannelongue Hospital, M3C, GHPSJ, University Paris-Saclay, Le Plessis-Robinson, France; Department of Pediatric and Congenital Heart Disease, Marie Lannelongue Hospital, M3C, GHPSJ, University Paris-Saclay, Le Plessis-Robinson, France

**Keywords:** Aortic arch surgery, Organ protection, Congenital

Deep hypothermia was considered a reliable method to reduce cerebral metabolic rate and is commonly used during surgeries requiring reconstruction of the aortic arch. During hypothermic antegrade selective cerebral perfusion, so-called regional cerebral perfusion (RCP), the autoregulation of the cerebral blood flow (CBF) is likely to be abolished [1], rendering the CBF of the neonate extremely susceptible to changes in perfusion pressure, acid–base disorders and interactions between the blood viscosity and microcirculatory flow. During RCP, the CBF is not measured directly; however, it is assumed that it is a function of the RCP rate and that the cerebral blood pressure is comparable to the pressure measured in the radial or brachial artery. This is further complicated by the fact that the RCP flow is actually delivered to both the brain and the right subclavian artery, and a variable rate is shunted through collaterals to the lower body via an extensive network of collateral arterial supply [2]. RCP performed in adequate flow during aortic arch reconstruction provides not only cerebral protection but also abdominal organ perfusion, indicated by postoperative low serum lactate concentration and other organ biomarkers [3]. The optimal temperature, flow rate, haematocrit and blood pressure of RCP for optimal brain protection, however, remain under debate. Although the RCP rate was set at 50 ml kg^−1^ min^−1^ when the technique was initially described [4], later work reported ASCP rates varying between 20 ml kg^−1^ min^−1^ [1, 5], 30 ml kg^−1^ min^−1^ [6], 40 ml kg^−1^ min^−1^ [7] and up to 94 ml kg^−1^ min^−1^ [8]. The variability of the collateral flow towards the abdominal organs could partly explain these wide variations. Since the autoregulation of the CBF is abolished, CBF is expected to vary according to the cerebral perfusion pressure. Older studies indicated that perfusion pressures of 20–25 mmHg were acceptable for the maintenance of cerebral blood volume [1] and resulted in good neuro-developmental outcomes at 1 year of age [7]. RCP at 20°C reported an abrupt decline of the CBF below 40 mmHg [9]. A mean arterial pressure objective of 40–50 mmHg is reported nowadays [10].

Among the surrogates of CBF that have been used for continuous monitoring of CBF, the regional cerebral oxygen saturation is a noninvasive metric which has been shown to be a sensitive indicator of rapid changes in CBF [11]. On the other hand, although linked with multiple parameters which determine the ratio between delivery and utilization of oxygen, it is acknowledged that over brief periods of time, CBF is the most potent determinant of regional cerebral oxygen saturation fluctuations [12]. To overcome the uncertainties around RCP rate and pressure, after cooling to 17–22°C, the adjustment of the flow rate to maintain cerebral rSO_2_ and Doppler flow velocity within 10% of the baseline recorded during full-flow bypass was proposed [8]. Nevertheless, 14 out of 34 patients had cerebral rSO_2_ of 95%, placing them at risk for cerebral hyperperfusion. Further investigations are needed to identify the target cerebral rSO_2_ during RCP.

The prevalence of kidney injury reported in the present study is comparable to that reported previously in neonates [13]. Despite the exponential drop in renal metabolism and renal oxygen consumption rate during hypothermia, which should have protected the kidneys here, the authors failed to reach a lower kidney injury rate with their high-rate perfusion technique. Experimental work demonstrated a >20% decrease in renal oxygen delivery 30 min after the onset of CPB despite optimal CPR rate and pressure parameters [14], suggesting that the cause of CPB-related kidney injury is CPB itself, and its main mechanism is ischaemia. Unfortunately, the puzzled records of renal rSO_2_ monitoring enables the authors to make objective conclusions regarding the renal perfusion during high-rate selective cerebral perfusion. However, rSO_2_ monitoring in the thigh is a potentially good surrogate of somatic perfusion, and here, the authors show a good correlation between low thigh rSO_2_ and postoperative kidney injury [15].
